# Factors Influencing Marker Expressions of Cultured Human Cord Blood-Derived Mast Cells

**DOI:** 10.3390/ijms241914891

**Published:** 2023-10-04

**Authors:** Shahrzad Alimohammadi, Kana Masuda-Kuroki, Attila Gábor Szöllősi, Anna Di Nardo

**Affiliations:** 1 Department of Dermatology, School of Medicine, University of California San Diego, La Jolla, CA 92037, USA; shalimohammadi@health.ucsd.edu (S.A.); kkuroki@health.ucsd.edu (K.M.-K.); 2 Doctoral School of Molecular Medicine, University of Debrecen, 4032 Debrecen, Hungary; szollosi.attila@med.unideb.hu; 3 Department of Immunology, Faculty of Medicine, University of Debrecen, 4032 Debrecen, Hungary

**Keywords:** human stem cells, CD34^+^ cord blood-derived mast cells, immune response, c-KIT, FcεRI, MRGPRX2, TLR2, tryptase, chymase

## Abstract

Mast cells (MCs) are tissue-resident immune cells of a hematopoietic origin that play vital roles in innate and adaptive immunity. Human MCs can be isolated and differentiated from various tissue sources, including cord blood, when supplemented with cytokines such as stem cell factor, interleukin 3, and interleukin 6. Our current research study has shown significant differences in the marker expressions of human cord blood-derived mast cells (hCBMCs) based on donor dependency and the type of medium used for culturing and differentiation. These findings are particularly relevant given the challenges of obtaining specialty media influencing MC phenotypic marker expressions. We found that hCBMCs cultured in StemSpanTM-XF medium had a moderate expression of mast/stem cell growth factor receptor Kit (c-KIT) (mRNA and protein), low expressions of FcεRI (mRNA) and TLR2 (mRNA and protein) but had high levels of MRGPRX2 (mRNA and protein) expressions. In contrast, hCBMCs cultured in Stem Line II medium expressed FcεRI and TLR2 (mRNA and protein) with higher c-KIT but had lower MRGPRX2 expressions compared to the hCBMCs cultured in the StemSpanTM-XF medium. These results suggest that it is crucial to consider both donor dependency and the medium when investigating MC functions and that further research is needed to fully understand the impact of these factors on the hCBMC marker expressions.

## 1. Introduction

Mast cells (MCs) are multifunctional immune cells that reside in tissues and are involved in various physiological processes such as tissue repair and wound healing, as well as immunity via protection against pathogens or inducing immune tolerance [1]. They play a significant role in both innate and adaptive immune responses associated with IgE, as they are the primary source of mediators responsible for severe and fatal IgE-dependent hypersensitivity, anaphylaxis, and acute allergic reactions [2,3]. MCs were initially believed to originate from pluripotent hematopoietic cells in the bone marrow; however, according to recent studies, they also originate from yolk sac-derived erythro-myeloid progenitors [4]. Immature MCs differentiate and develop into mature MCs in the presence of stem cell factor (SCF) and interleukin 3 (IL-3). SCF is crucial for developing and expanding MC progenitor cells expressing CD34^+^ antigen and c-KIT receptor, while IL-3 cytokine is responsible for their differentiation and maturation [1,5,6,7]. During differentiation, the MC progenitors’ morphology sequentially change [8]. Then, MCs migrate into most vascularized tissues and complete their maturation, although matured MCs reside near nerves, vessels, smooth muscles, gastrointestinal (GI) tract, and mucus-producing glands. According to murine models, in the early developmental stage, yolk sac-derived MCs may migrate to various connective tissues, including the skin. However, tissue MCs can also derive from hematopoietic tissues, namely the bone marrow, while the precursors circulate prior to further maturation in peripheral tissues [2].

MC populations are distinct by proteases in the granules, as one population may possess chymase and tryptase, while another population may have only tryptase and no chymase [2]. Tryptase and chymase are the most substantial serine proteases present in the MC granules. They are shown to be involved in extracellular matrix degradation, atherosclerosis, cardiovascular disease, and metabolic disorders [9,10]. By hydrolyzing chemokines and cytokines, tryptase and chymase modulate immune responses and battle inflammation by neuropeptide and allergen inactivation [11]. Hence, human MCs can be classified based on their protease content. For instance, human lung MCs contain chondroitin sulfate proteoglycans and heparin, while human dermal MCs express both tryptase and chymase [12].

When activated, MCs produce a broad range of cytokines and cytoplasmic granule-associated mediators, namely histamine, proteases, and metalloproteinases, in addition to lipid mediators such as prostaglandins (PGs), cysteinyl leukotrienes (cysLTs), and other de novo synthesized chemokines and cytokines [3]. Skin-derived primary MCs can be isolated using both mechanical and proteolytic digestion processes. However, the number of skin MCs is limited, and these isolation methods may result in impure or nonviable cells, which could also be activated during this process. Additionally, the study of mast cells is limited since MC cell lines may not express specific functional receptors [13]. There is a high demand for sources of human progenitor cells that can be directed to acquire specific tissue characteristics and be available in sufficient quantities for functional and pharmacological studies. Mast cell progenitors derived from umbilical cord blood and cord matrix can be separated magnetically and grown under the influence of stem cell factor, interleukin-6, interleukin-3, and other cytokines to create distinct mast cell populations. By expanding and differentiating stem cells into mast cells, researchers can obtain a larger number of mast cells, improving mast cell research. Due to these advantages, CD34+ human cord blood mast cells are recognized as a highly qualified source of human mast cells.

To overcome these challenges, CD34^+^ human cord blood-derived stem cells (hCBMCs) are known as a well-qualified source to culture MC populations in higher yields while minimizing unwanted changes during the culture process [14].

Mature MCs express several functionally important receptors, such as mast/stem cell growth factor receptor kit (c-KIT) and high-affinity immunoglobulin epsilon receptor alpha-subunit (FcεRIα), as their characteristic functional markers [14,15]. Besides c-KIT and FcεRIα, hCBMCs also express the MAS-related G protein-coupled receptor X2 (MRGPRX2), which is responsible for histamine release when activated by neuropeptides and other cationic peptide stimulants [15]. In IgE-dependent immune responses, the crosslinking of Fc*ε*RI/IgE results in the activation of MCs together with the degranulation of a broad range of bioactive products, including histamine [16]. The IgE-induced activation of MCs results in the activation of epithelial, endothelial, smooth muscle, neurons, and other immune cells via inflammatory cell influx. Other than various cell activations, the existence of specific IgE in serum is a characteristic key of allergic asthma [12].

Pattern recognition receptors (PRRs) are involved in the nonspecific detection of foreign pathogens. Toll-like receptors (TLRs) are a subclass of PPRs that play a role in innate immunity and inflammatory responses [17]. Of the TLRs, TLR2 is essential due to its high affinity for bacterial lipoproteins, namely lipoteichoic acid (LTA) and peptidoglycan (PGN) [18]. According to many studies, in MCs, most TLRs, including TLR2, are expressed [19,20], although their expression levels vary among studies [21], with some only detectable at the mRNA level [3]. Other studies on bone marrow-derived MCs state that they only express a truncated version of the TLR2 receptor, lacking an intracellular signaling domain [20]. Given the importance of MCs’ response to TLRs and the abovementioned findings, careful regulation of the TLR2 expression is necessary.

MCs also respond to TLR agonists by secreting lipid mediators, cytokines, and chemokines, which would have profound downstream effects on the immune system. At the same time, TLR ligation itself provokes MCs’ response to antigens and terminates in cell sensitization via FcεRIα [20].

Although hCBMCs are commonly utilized for exploring MC functions, various differentiation methods are employed, and several media are available on the market. Due to their proprietary nature, their formulations are typically unknown. Availability of such specialty media adds to the challenges of relying on donors for these unique cells. This has frequently forced researchers to use different media, leading to concerns about the comparability and reproducibility of the obtained data.

Previous studies discovered that stem cell media can impact the growth, phenotype, function, marker expression level, and granule content of MCs derived from peripheral blood of CD133^+^ donors. Derakhshan et al. found that StemSpan media supplemented with serum resulted in higher c-KIT expression in cultured stem cells compared to other tested media [22,23,24]. Since different studies have shown varying marker expression in human MC models, we aimed to investigate whether using different media can affect the phenotype and functional traits of hCBMCs beyond donor dependency. To achieve our objective, we utilized two stem cell media—StemSpanTM-XF (medium A) and Stem Line II (medium B)—to culture hCBMCs and compared the outcomes. We observed differences in the marker expression between the two media and decided to conduct a more comprehensive side-by-side comparison. Our research has indicated that the expression levels of markers in hCBMCs can significantly vary among different donors. Additionally, the media used during differentiation, culture, and maturation of CD34^+^ hCBSCs can heavily influence the presence and levels of these markers. Therefore, it is crucial to consider these factors when working with hCBMCs to ensure accurate and reproducible results.

## 2. Results

### 2.1. Characteristic Markers of hCBMCs Vary between Donors

**Figure 1:**
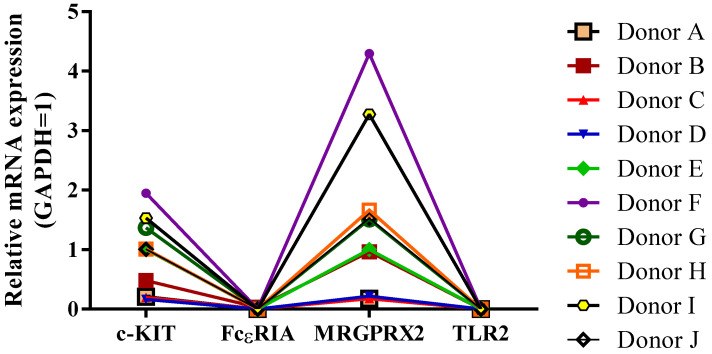
hCBMC-characteristic marker expression is donor-dependent, and stem cell medium highly alters these markers’ presence and/or absence. mRNA expression levels for c-KIT, FcεRIα, MRGPRX2, and TLR2 vary in hCBMCs differentiated from ten individuals (donors A–J, n = 10).

First, we investigated the mRNA expression of hCBMC characteristic markers differentiated from ten different individuals in medium A. The hCBMCs cultured from distinct CD34^+^ cells express a broad range of MC characteristic markers including c-KIT, FcεRIα, MRGPRX2, as well as TLR2 (Figure 1). Moreover, MRGPRX2 showed the highest difference in the mRNA expression among donors, while c-KIT showed lower mRNA expression variability than MRGPRX2. Interestingly, no mRNA expression was detected for either FcεRIα or TLR2 in all ten investigated donors. 

**Figure 2:**
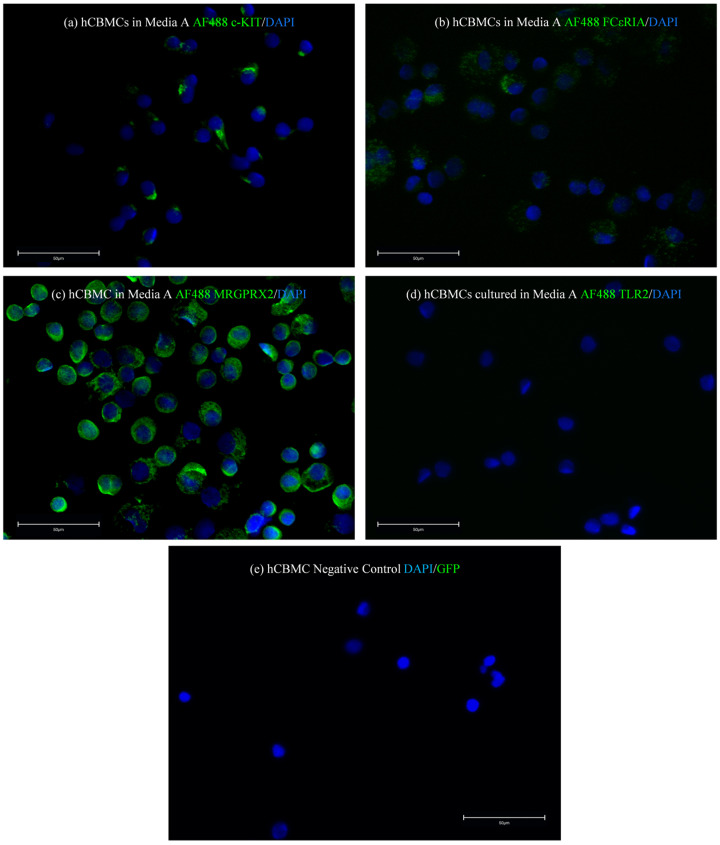
Representative photomicrographs of hCBMCs cultured in medium A. Immunofluorescence (IF) staining for c-KIT (**a**), FcεRIα (**b**), MRGX2 (**c**), TLR2 (**d**), and negative control where mast cells were only stained with DAPI, while the photo represents merged DAPI and GFP channels (**e**). All proteins, except TLR2, were stained with AF488-conjugated secondary antibodies, while TLR2 was AF488-conjugated. Medium A, StemSpan™-XF; medium B, Stem Line II; negative CTRL, hCBMCs stained with DAPI only as the negative control; DAPI, four′,6-diamidino-2-phenylindole.Blue fluorescence represents DAPI and green fluorescence represents AF488-conjugated antibody (GFP), Scale bar = 50 µm.

After evaluating the mRNA expressions of hCBMCs cultured in medium A, we investigated if protein expressions of c-KIT, FcεRIα, MRGPRX2, and TLR2 on hCBMCs correlated with our mRNA results. We cultured hCBMCs in medium A, and once they were fully matured, we stained them with unconjugated monoclonal anti-human c-KIT (Figure 2a), FcεRIα (Figure 2b), and MRGPRX2 (Figure 2c) primary antibodies, and AF488-conjugated TLR2 antibody (Figure 2d). AF488-conjugated antibodies were used as the secondary antibody for c-KIT, FcεRIα, and MRGPRX2 (green fluorescence). Based on our immunofluorescence (IF) results, hCBMCs cultured in medium A revealed the highest expression for MRGPRX2, moderate to low expression level for c-KIT, and common expression for FcεRIα. At the same time, there were no protein expressions for TLR2 similar to their mRNA expressions (Figure 2).

### 2.2. Stem Cell Media Influence the Marker Levels of mRNA and Protein Expressions in hCBMCs 

**Figure 3:**
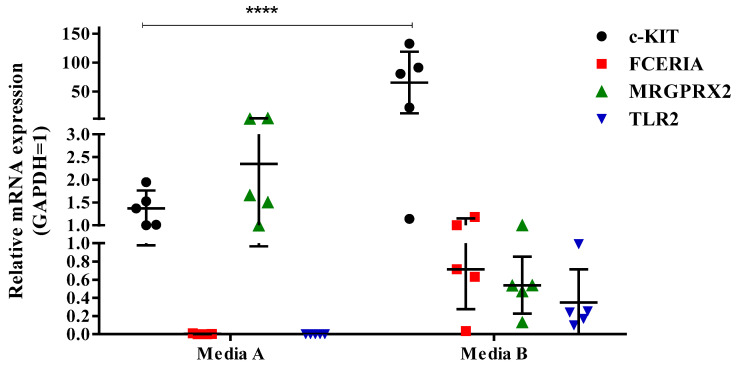
hCBMC marker expression varies when cultured in two different media. RT-qPCR-analyzed data are representative of triplicated experiments of five donors. Data shown are the means ± SD; **** *p* < 0.0001; medium A, StemSpan™-XF; medium B, Stem Line II.

After confirming the effect of media on the hCBMC marker protein expressions (Figure 2), we investigated the mRNA expression of five different donors cultured in two other media, media A and B. We conducted RT-qPCR to analyze the mRNA expression of c-KIT, FcεRIα, MRGPRX2, and TLR2 (Figure 3). These results indicate that the hCBMCs differentiated in medium A expressed c-KIT and MRGPRX2 but not FcεRIα and TLR2 at the mRNA level, confirming our results from Figure 1. However, the hCBMCs cultured in medium B showed higher c-KIT, FcεRIα, and TLR2 mRNA expressions, while the mRNA expression of MRGPRX2 was decreased. Based on these findings, utilization of different media can result in changes in both the marker expressions and levels of hCBMCs.

Besides the MC markers mentioned above, the mRNA expression level of chymase (CMA1) was minimal and comparable in the hCBMCs cultured in medium A compared to medium B in five distinct donors (Supplementary Figure S1). However, the same donors expressed higher levels of tryptase (TPSB2) at the gene level when cultured in medium B compared to medium A (Supplementary Figure S1).

**Figure 4:**
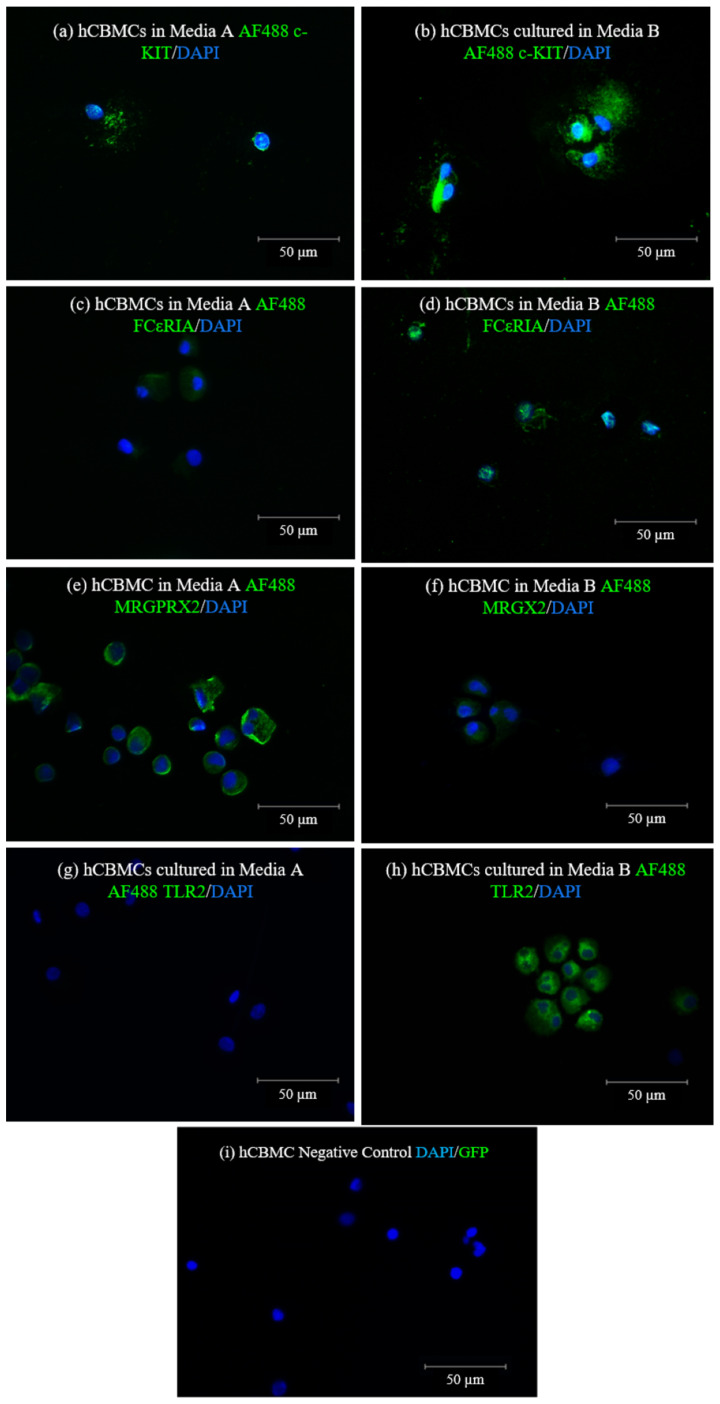
Representative photomicrographs of hCBMCs cultured in two different media. Immunofluorescence (IF) staining for c-KIT (**a**,**b**), FcεRIα (**c**,**d**), MRGX2 (**e**,**f**), and TLR2 (**g**,**h**) in medium A (left panels) or medium B (right panels), (**i**) hCBMCs stained with DAPI only as the negative control where mast cells were only stained with DAPI, while the photo represents merged DAPI and GFP channels. All proteins except TLR2 were stained with AF488-conjugated secondary antibody, while TLR2 was AF488-conjugated. Medium A, StemSpan™-XF; medium B, Stem Line II; DAPI, four′,6-diamidino-2-phenylindole. Blue fluorescence represents DAPI and green fluorescence represents AF488-conjugated antibody (GFP), Scale bar = 50 µm.

After confirming the influence of the medium on the mRNA expression levels in hCBMCs (Figure 3), to verify if these differences are present at the protein level, immunofluorescence staining (IF) was performed (Figure 4a–h). According to IF images, the hCBMCs cultured in medium A presented lower protein expression for c-KIT (Figure 4a,b), higher expression of MRGPRX2 (Figure 4c,d), and no expression of FcεRIα and TLR2 compared to the same hCBMCs batch cultured in medium B. Based on the findings, it has been confirmed that both media A and B (as depicted in Figure 4) have the same impact on hCBMCs at the protein level as on the mRNA expression levels.

## 3. Discussion

MCs are crucial in the body’s immune response and play significant roles in innate and adaptive immune systems. MCs originate from extraembryonic yolk sacs and can be differentiated from bone marrow and blood-derived human stem cells (HSCs). Heterogeneity among MCs in their resident tissues affects the protease expression [14]. MCs express a variety of characteristic receptors, including c-KIT, FcεRIα, and MRGPRX2, as well as TLRs [14,18].

It remains challenging to investigate MC heterogeneity in different tissues and organs despite the numerous published studies that have enhanced MC research and identified essential roles for MCs and their signaling pathways in response to pathogens. Therefore, exploring similarities and dissimilarities is critical to understanding MCs’ functional distinctness [20].

According to the literature, there is a broad range of variability in the marker expression levels in MC studies. In addition to the differences in species types (e.g., murine and human), MCs present variations in the marker expression in primary cultures, cell lines, and residential tissues. Besides the location, the source of these cells (e.g., bone marrow, peritoneal, peripheral blood, cord blood) plays crucial roles in the resulting variability in the MC phenotype when grown in vitro. These nonuniformities in the marker expressions highly impact the progress in studying MCs. Monitoring possible factors that implement these alterations is pivotal to overcoming this challenge. 

MCs express a wide range of markers unique to their anatomical locations, and recent studies categorized MCs into seven different subtypes based on their diverse marker expressions [20].

A crucial question about MCs that needs to be addressed is whether these variable marker expressions indicate exclusive biological capabilities and functions [10]. Based on our findings in this study, donor dependency plays a fundamental role in the expression levels of hCBMC-specific markers, which typically makes it challenging to determine further changes upon treatments. To address this concern, investigating multiple donors as potential sources of CD34^+^ HSCs can lead to more reliable results, which naturally results in increased costs. This donor dependence is a well-known and documented challenge of working with primary cells and is typically factored into research dealing with such cells.

In addition to donor dependency, the medium in which hCBMCs differentiate from CD34^+^ HSCs also alters their marker expression irreversibly. Previous studies state that stem cell media change the MCs phenotype and development [8]. Thus, it is difficult to obtain specialty media, which adds to the challenges of relying on donors for these unique cells. This has forced researchers to use different media, leading to concerns about comparability. There are numerous medium options for HSCs that are available in the market. However, their formulations are not disclosed due to their proprietary nature. It is essential to choose and use appropriate media depending on the marker of interest, as it forms the core of the study. The effect of these specialty media on the MCs is much less well-documented than the effect of donor dependence and presents an essential gap in our collective knowledge.

In this study, we differentiated and cultured hCBMCs in two different stem cell media available at the time. We realized that each of these media can help in different fields of interest in the MC marker expressions and drive MCs to express a specific type of receptor while causing the downregulation of others. Our results revealed that the hCBMCs cultured in medium A failed to express FcεRIα and TLR2 at the mRNA level. Additionally, they had low FcεRIα and no TLR2 protein expressions. As reported by previous studies, both FcεRIα and TLR2 markers are shown to be expressed on hCBMCs, both at gene and protein levels. Based on these findings and our q-PCR results, we decided to investigate if the medium change could have been responsible for the difference in the mRNA expression of the mentioned markers in hCBMCs.

In contrast to low FcεRIα and no TLR2 expression, the hCBMCs cultured in medium A expressed high levels of MRGPRX2 with a moderate c-KIT expression at both the mRNA and protein levels. On the other hand, the hCBMCs cultured in medium B expressed FcεRIα and TLR2. At the same time, the MRGPRX2 expression was lower than in medium A, while the c-KIT mRNA and protein expressions were induced in medium B. In addition, while the mRNA expression level of chymase was comparable in medium A and B, the gene expression of tryptase was slightly higher in medium B compared to medium A. 

In light of the findings of this study, we concluded that regarding culturing hCBMCs from CD34^+^ HSCs, the expression of MCs characteristic markers varies in different donors, possibly due to their gender and hereditary pathological disorders, in addition to possible maternal medications and treatments during pregnancy. Furthermore, the stem cell medium in which hCBMCs are cultured alters their marker expression and levels, resulting in different MC phenotypes. Thus, the choice of the medium is critical in in vitro MC research. To choose a suitable medium for the phenotype of interest, one must consider if the study of interest is focused on atopic asthma, asthma, allergy, perennial allergic rhinitis, seasonal allergic rhinitis, or viral reactions, where testing the c-KIT, IgE, and TLR2 expressions is crucial, culturing hCBMCs in medium B is preferred and recommended compared to medium A. On the contrary, if the focus of the study is neuropeptides, drug response, or chronic urticaria where MRGX2 is significantly increased, medium A should be considered the appropriate medium compared to medium B. 

As mentioned in previous studies, various factors including age, gender and pathological disorders influence heterogeneity in MC populations. MCs, similar to other cell populations, undergo age-related modifications such as in number, localization, and activation throughout their lifetime, which adversely affects the etiology and progression of many physiological conditions including pathological diseases [25]. Other than age, gender plays a crucial role in differences in adult MCs, as female MCs express a highly different RNA transcriptome compared to male MCs, and display an enhanced capacity to store mediators, namely proteases and histamine, within their secretory granules [26]. To summarize, the expression levels of hCBMC markers are significantly influenced by donor dependency due to the heterogeneity in age, gender, and pathological conditions. Other than that, microenvironmental factors, particularly the type of the HSC medium, impact the mast cells’ phenotypic marker expression and their levels. Therefore, it is crucial to consider both factors when studying hCBMCs and reporting in the Material and Methods section (Section 4). This will ensure a more accurate understanding of the markers and MCs phenotype of interest.

## 4. Materials and Methods

### 4.1. Human MC Culture

Human CD34^+^ cells were isolated from human cord blood under the Institutional Review Board (IRB): Project #190445X, the University of California San Diego human research protection program. To isolate mononuclear cells, the umbilical cord blood of donor was diluted with PBS—Phosphate-buffered saline (Gibco, Waltham, MA, USA, Cat. No 10010031) in 1:1 ratio followed by Ficoll (Cytiva, Marlborough, MA, USA, Cat. No 17144002) density gradient centrifugation at 400× *g* centrifugation force for 15 min at room temperature with low centrifuge brake. The mononuclear cell ring was carefully removed from the interphase and washed three times with MACS buffer containing 0.5% BSA—bovine serum albumin (GeminiBio, Sacramento, CA, USA, Cat. No SKU# 700-100P-100) and two mM EDTA—Ethylenediaminetetraacetic acid (Invitrogen, Waltham, MA, USA, Cat. No AM9260G) in PBS, pH 7.2, at 2−8 ° C. Remaining red blood cells were lysed using ACK lysing buffer (Thermo Fisher, Waltham, MA, USA, Cat. No A1049201) for 5 min at room temperature. Further, CD34^+^ HSCs were separated from other mononuclear cells using immunomagnetic positive selection EasySep™ Human CD34 Positive Selection Kit II (STEMCELL TechnologiesTM, Vancouver, BC, Canada, Cat. No 17896) followed by three washes with cold MACS buffer. Resulting CD34^+^ HSC cells were then cultured in two different serum-free media, medium A, xeno-free StemSpanTM-XF medium (STEMCELL TechnologiesTM, Vancouver, BC, Canada, Cat. No 100-0073) and medium B, Stem Line II (Sigma Aldrich, St. Louis, MO, USA, Cat. No S0192). Both media were supplemented with 1% antibiotic-antimycotic (Thermo Fisher, Waltham, MA, USA, Cat. No 15240096), recombinant human stem cell factor (rhSCF 100 ng/mL, R&D Systems, Minneapolis, MN, USA, Cat. No 255-SC-200/CF), recombinant human IL-6 from week 5 (rhIL-6 100 ng/mL, R&D Systems, Minneapolis, MN, USA, Cat. No 206-IL-200/CF), and recombinant human IL-3 (rhIL-3 20 ng/mL, R&D Systems, Minneapolis, MN, USA, Cat. No 203-IL-100/CF, first week only). On week 10, mature hCBMCs were consistently generated, as confirmed by the expression of c-KIT (CD117) and FcεRIα (IgE) maturation markers (TaqMan Assays Hs00174029 m1 for c-KIT and Hs00758600 m1 for FcεRIα (IgE), both from Thermo Fisher, Waltham, MA, USA, Cat. No 4453320 and 4448892, respectively). 

### 4.2. Real-Time Quantitative RT-qPCR

After week 10 of CD34^+^ culture with the mentioned cytokine cocktail in the human mast cells culture section, mature hCBMC cells were collected, and total RNA was isolated by Quick-RNA Miniprep Kit (Zymo Research, Irvine, CA, USA, Cat. No R1054). A minimum of 1000 ng/µL RNA concentration was used for cDNA synthesis by the Applied Biosystems™High-Capacity cDNA Reverse Transcription Kit (Applied Biosystems, Waltham, MA, USA, Cat. No 4368814) according to the manufacturer’s instructions. A real-time polymerase chain reaction (PCR) instrument performed real-time quantitative polymerase chain reaction (RT-qPCR), and thermal cycler devices were used to perform reverse transcription and qPCR (Bio-Rad, Hercules, CA, USA, and Applied Biosystems, Waltham, MA, USA, respectively). TaqMan Fast Advanced Master Mix (Applied Biosystems, Waltham, MA, USA, Cat. No 4444557) was used in all qPCR experiments. TaqMan Assays Hs00174029 m1 for c-KIT, Hs00758600 m1 for FcεRIα (IgE), Hs00365019 s1 for MRGPRX2, and Hs00152932 m1 for TLR2, Hs00156558 m1 for CMA1, Hs02576518_gH for TPSB2 were all purchased from Thermo Fisher (Thermo Fisher Scientific, Waltham, MA, USA, Cat. No. 4453320, 4448892, 4453320, and 4453320, respectively). Raw gene expression data were analyzed with the 2^−ΔΔCT^ (Cycle Threshold) method to determine the quantification of expression levels. Genes of interest were normalized to Glyceraldehyde 3-phosphate dehydrogenase (GAPDH) as the housing gene, as well as their fold change relative to GAPDH (Thermo Fisher Scientific, Waltham, MA, USA, Cat. No. 4325792) expression in baseline controls. The number of biological donors was n = 10 for Figure 1 and n = 5 for Figure 3 and Supplementary Figure S1. Each experiment was performed more than two times. The presented values are expressed as mean ± standard deviation.

### 4.3. Immunofluorescence (IF) of hCBMCs 

Matured hCBMCs were collected and fixed with 1% paraformaldehyde (Thermo Scientific, Waltham, MA, USA, Cat. No. 043368.M) in PBS—Phosphate-buffered saline (Gibco, Waltham, MA, USA, Cat. No 10010031) for 10 min at room temperature, followed by washing three times in fresh PBS. Fixed hCBMCs were then permeabilized with 0.5% Triton X-100 (Sigma, St. Louis, MO, USA, Cat. No X100-100 mL) in PBS for 10 min at room temperature, followed by three times washing in PBS. Fixed and permeabilized hCBMCs were stained overnight at four °C with unconjugated monoclonal anti-human c-KIT antibody (Cell Signaling, Danvers, MA, USA Cat. No 3074T), unconjugated monoclonal anti-human FcεRIα (BioLegend, San Diego, CA, USA, Cat. No 334602), unconjugated polyclonal anti-human MRGPRX2 antibody (Thermo Scientific, Waltham, MA, USA, Cat. No PIPA5113198), and Alexa Fluor^®^ 488-conjugated monoclonal anti-human CD282 (TLR2) antibody (Invitrogen, Waltham, MA, USA, Cat. No MA1-40320). After three times washing with PBS after primary antibody staining, hCBMCs were stained with secondary AF488-conjugated F(ab’)2-Goat anti-rabbit IgG and AF488-conjugated F(ab’)2-Goat anti-mouse IgG antibodies (Invitrogen, Waltham, MA, USA, Cat. No A-11070 and A-11001, respectively) for 1 h at room temperature. The hCBMCs were then washed three times with PBS for 5 min and mounted in ProLong™ Gold Antifade Mounting with DNA Stain DAPI (Invitrogen, Waltham, MA, USA, Cat. No P36935). Fluorescence images were taken with an EVOS fluorescent microscope (EVOS M5000, Invitrogen, Waltham, MA, USA, Cat. No AMF5000). Antibody dilutions for unconjugated primary unconjugated c-KIT, FcεRIα, and MRGX2 and Alexa Fluor^®^ 488-conjugated monoclonal anti-human TLR2 antibodies were 1:50. The secondary AF488-conjugated F(ab’)2-Goat anti-rabbit IgG and AF488-conjugated F(ab’)2-Goat anti-mouse IgG antibodies were diluted 1:500 with PBS used as the dilution buffer in all staining cases. 

### 4.4. Statistical Analysis

GraphPad Prism version 7 (GraphPad Software, Boston, MA, USA) was used to prepare figures and analysis. One- or two-way analysis of variance (ANOVA) and Tukey’s tests were applied to analyze the differences among more than two groups with a confidence level of 95%.

## 5. Conclusions

In in vitro MC research, donor dependency and the medium are two key factors influencing the hCBMC marker expression at the mRNA and protein levels.

## Data Availability

The supporting data of this study are available from the corresponding author upon request.

## Supplementary Materials

The following supporting information can be downloaded at https://www.mdpi.com/article/10.3390/ijms241914891/s1.
