# Supplementary material for: Factors Influencing Marker Expressions of Cultured Human Cord Blood-Derived Mast Cells
Source: Int J Mol Sci. 2023 Oct 4;24(19):14891. doi: 10.3390/ijms241914891 (PMC10573221; doi:10.3390/ijms241914891)
Supplement: Supplementary file 1 [file ijms-24-14891-s001.zip › Supplementary figure S1.pdf]

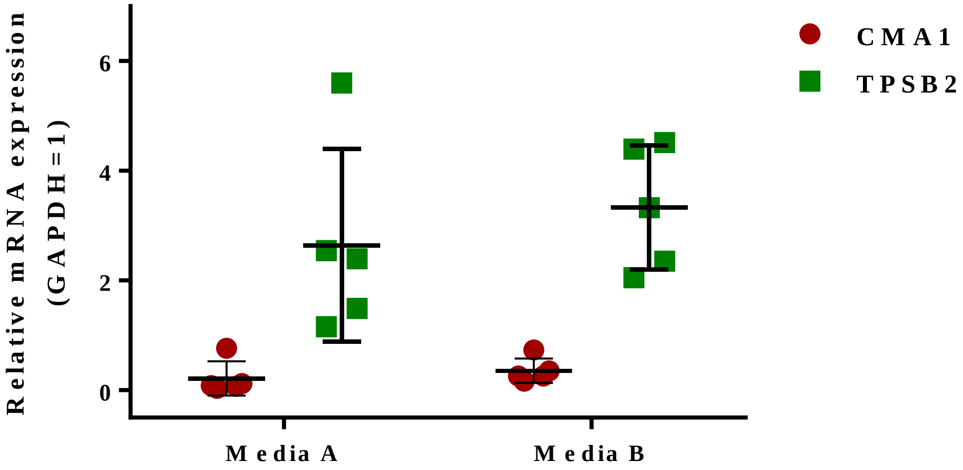

Supplementary Figure S1. hCBMCs marker expression varies when cultured in two different media. RT-qPCR ana-lyzed data is representatives of triplicated experiments of five donors. Data shown are the means ± SD. Media A, StemSpan™-XF;Media B, Stem Line II.
